# Prognostic value of pretreatment modified Glasgow Prognostic Score in small cell lung cancer: A meta-analysis

**DOI:** 10.1097/MD.0000000000035962

**Published:** 2023-11-10

**Authors:** Yulian Xie, Hongjun Li, Yang Hu

**Affiliations:** a Lung Cancer Center, West China Hospital, Sichuan University/West China School of Nursing, Sichuan University, Chengdu, P.R. China; b Department of Thoracic Surgery, China Hospital, Sichuan University, Chengdu, P.R. China.

**Keywords:** meta-analysis, modified Glasgow Prognostic Score, prognosis, small cell lung cancer

## Abstract

**Background:**

The prognostic role of pretreatment modified Glasgow Prognostic Score (mGPS) in small cell lung cancer (SCLC) patients remains unclear now.

**Methods:**

The PubMed, EMBASE, Web of Science, and CNKI electronic databases were searched up to December 14, 2022. The primary and secondary outcomes were overall survival and progression-free survival, respectively. The hazard ratios (HRs) and 95% confidence intervals (CIs) were combined to assess the association between pretreatment mGPS and survival of SCLC patients. Subgroup analysis based on the country, tumor stage, treatment and comparison of mGPS were further conducted and all statistical analyses were performed by STATA 15.0 software.

**Results:**

A total of ten retrospective studies involving 2831 SCLC patients were included. The pooled results demonstrated that elevated pretreatment mGPS was significantly related to poorer overall survival (HR = 1.90, 95% CI: 1.36–2.63, *P* < .001) and progression-free survival (HR = 1.40, 95% CI: 1.13–1.74, *P* = .002). Subgroup analysis stratified by the country, tumor stage, treatment and comparison of mGPS also showed similar results.

**Conclusion:**

Pretreatment mGPS was significantly associated with prognosis in SCLC and patients with elevated mGPS experienced obviously worse survival. Thus, pretreatment mGPS could serve as a novel and reliable prognostic indicator in SCLC patients.

## 1. Introduction

Small cell lung cancer (SCLC) accounts for about 15% of all lung cancer cases.^[1]^ It is characterized by high degree of malignancy, rapid progression and metastasis, and poor prognosis.^[2–4]^ The tumor stage of SCLC patients is usually evaluated according to the Veterans Administration Lung Study Group staging system which includes limited stage SCLC and extensive stage SCLC and about 70% of the patients are already in the extensive stage at the time of diagnosis.^[2,5]^ In the past decades, there has been no significant progress in the targeted therapy and immunotherapy of SCLC and most patients could only be treated by traditional chemotherapy and radiation.^[6–8]^

The relapse rate of SCLC after first-line treatment is certainly high and it is believed that the current staging system is not sufficient to accurately evaluate survival of SCLC patients.^[9,10]^ Therefore, a lot of indicators which might contribute to the prediction of survival of SCLC patients have been reported in the last several years such as the sarcopenia^[11]^ and lung immune prognostic index (LIPI).^[12]^ However, these indexes are limited in clinics due to the inconvenience and low efficiency. Then, some indicators based on laboratory examinations have been widely described such as the albumin to globulin ratio, neutrophil to lymphocyte ratio, lymphocyte to monocyte ratio and d-dimer concentration.^[13–16]^ Unfortunately, these continuous indicators are unstable and there are no relatively objective criteria for clinical application.

Modified Glasgow Prognostic Score (mGPS) is calculated based on peripheral C-reactive concentrations and albumin levels with a clear score criteria: C-reactive protein level ≤ 10 mg/L and albumin level ≥ 35 g/L are defined as mGPS 0, C-reactive protein level > 10 mg/L and albumin level ≥ 35 g/L are defined as mGPS 1 and C-reactive protein level > 10 mg/L and albumin level < 35 g/L are defined as mGPS 2.^[17]^ Up to now, its prognostic value has been verified by the systematic review and meta-analysis in several types of cancers including the urothelial carcinoma, ovarian cancer, pancreatic cancer, renal cell carcinoma, and gynecologic cancer.^[18–22]^ As for lung cancer, Jin et al conducted a meta-analysis to verify prognostic value of mGPS in lung cancer after including 11 studies in 2017.^[23]^ However, they did not well identify the prognostic role of pretreatment mGPS in SCLC and the association between pretreatment mGPS and survival of SCLC patients remains unclear now.

Therefore, the aim of this meta-analysis was to further identify the prognostic value of pretreatment mGPS in SCLC, which might contribute to the survival prediction and therapy strategy formulation.

## 2. Materials and methods

This meta-analysis was conducted according to the Preferred Reporting Items for Systematic Reviews and Meta-Analyses guidelines (2020).^[24]^

### 2.1. Literature search

The PubMed, EMBASE, Web of Science and CNKI databases were searched from inception to December 14, 2022 in this meta-analysis. During the search, the following key words were used: modified Glasgow Prognostic Score, mGPS, small cell lung cancer, SCLC, prognosis, prognostic, and survival. The specific search strategy was as follows: (modified Glasgow Prognostic Score OR mGPS) AND (small cell lung cancer OR SCLC) AND (prognosis OR prognostic OR survival). To avoid omissions, the MeSH terms and free words were applied and references cited in included studies were also reviewed.

### 2.2. Inclusion and exclusion criteria

The inclusion criteria were as follows: (1) patients were pathologically diagnosed with SCLC; (2) the C-reactive protein and albumin levels were detected before antitumor treatments and the mGPS was classified according to the definition: C-reactive protein level ≤ 10 mg/L and albumin level ≥ 35 g/L were defined as mGPS 0, C-reactive protein level > 10 mg/L and albumin level ≥ 35 g/L were defined as mGPS 1 and C-reactive protein level > 10 mg/L and albumin level < 35 g/L were defined as mGPS 2;^[17]^ (3) patients were divided into different groups based on the mGPS and the overall survival (OS) and (or) progression-free survival (PFS) were compared between groups; 4) the hazard ratios (HRs) and 95% confidence intervals (CIs) were reported in articles, or the Kaplan–Meier survival curves were provided.

The exclusion criteria were as follows: (1) duplicated or overlapped data; (2) meeting abstracts, letters, editorials, reviews or animal trials; (3) insufficient information to assess the methodological quality.

### 2.3. Data extraction

In this meta-analysis, the following information was collected from each included studies: the name of first author, publication year, country, sample size, tumor stage, treatment, comparison of mGPS, endpoints, HR, and corresponding 95% CI.

### 2.4. Methodological quality assessment

Due to retrospective nature of all included studies, the Newcastle-Ottawa Scale (NOS) score tool consisting of selection, comparability and outcome was applied to evaluate the quality of included studies and studies with a NOS score of 6 or higher were regarded as high-quality studies.^[25]^

The literature search, selection, data collection, and quality assessment were all performed by 2 authors independently and all disagreements were resolved by team discussion.

### 2.5. Statistical analysis

All statistical analyses were conducted by STATA 15.0 software. The HRs with 95% CIs were combined to explore the association between pretreatment mGPS and OS and PFS of SCLC patients. The heterogeneity among the included studies was evaluated by *I*^2^ statistics and *Q* tests. If obvious heterogeneity was detected presenting as *I*^2^ >50% or *P* value <0.1, the random-effects model was used; otherwise, the fixed-effects model was applied.^[26]^ Subgroup analysis based on the country (China vs Japan), tumor stage (mixed vs extensive stage), treatment (mixed vs chemotherapy) and comparison of mGPS (0 vs 1; 0 vs 2) were further performed. Besides, sensitivity analysis was conducted to detect the source of heterogeneity and assess the stability of pooled results. Furthermore, Begg funnel plot and Egger test were conducted to detect publication bias.^[27,28]^ Significant publication bias was defined as *P* < .05.

## 3. Results

### 3.1. Literature search and selection process

**Figure 1.**
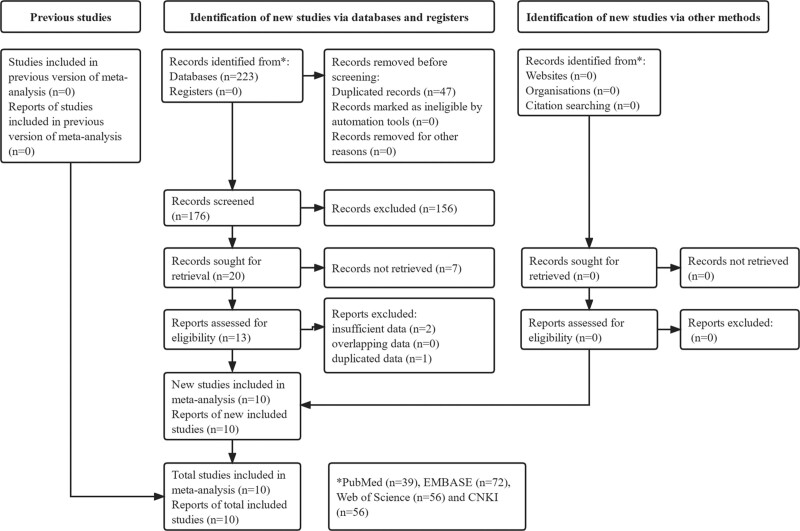
The flow diagram of this met-analysis.

Initially, 223 records were identified from the 4 databases and 47 duplicated records were removed. Then the 156 and 7 publications were excluded after reviewing the titles and abstracts, respectively. The full texts of remaining 13 publications were carefully reviewed and 10 studies were included in this meta-analysis eventually.^[29–38]^ The detailed selection process was presented in Figure 1.

### 3.2. Basic characteristics of included studies

In overall, 2831 SCLC patients were enrolled in the analysis. With the exception of study by Winther-Larsen et al,^[38]^ all other studies were from China or Japan. The sample size ranged from 41 to 890 and most patients received the chemotherapy. Besides, all included studies were retrospective and high-quality studies with a NOS score ≥ 6. Specific characteristics were shown in Table 1.

**Table 1:** Basic characteristics of included studies.

Author	Year	Country	Sample size	Stage	Treatment	Comparison of mGPS	Endpoint	NOS
Zhou^[29]^	2015	China	359	Limited + extensive	Chemotherapy + others	0 vs 1, 0 vs 2	OS	7
Kurishima^[30]^	2017	Japan	332	Limited + extensive	Chemotherapy + others	0 vs 1, 0 vs 2	OS	7
Minami^[31]^	2017	Japan	97	Extensive	Chemotherapy	0/1 vs 2	OS, PFS	7
Sonehara^[32]^	2019	Japan	83	Extensive	Chemotherapy + others	0 vs 1, 0 vs 2	OS	7
Zhou^[33]^	2019	China	451	Limited + extensive	Chemotherapy	0 vs 1, 0 vs 2	OS	6
Zhan^[34]^	2019	China	277	Limited + Extensive	Chemotherapy	0 vs 1 vs 2	OS, PFS	8
Igawa^[35]^	2020	Japan	41	Extensive	Chemotherapy	0 vs 1/2	OS, PFS	6
Mao^[36]^	2021	China	118	Limited + extensive	Chemotherapy	0 vs 1 vs 2	OS	7
Zhang^[37]^	2021	China	183	Limited + extensive	Chemotherapy	0 vs 1 vs 2	OS	8
Winther-Larsen^[38]^	2022	Denmark	890	Limited + extensive	NR	0 vs 1, 0 vs 2	OS	6

mGPS = modified Glasgow Prognostic Score, NOS = Newcastle-Ottawa Scale, NR = not reported, OS = overall survival, PFS = progression-free survival.

### 3.3. The association between pretreatment mGPS and OS in SCLC patients

**Figure 2.**
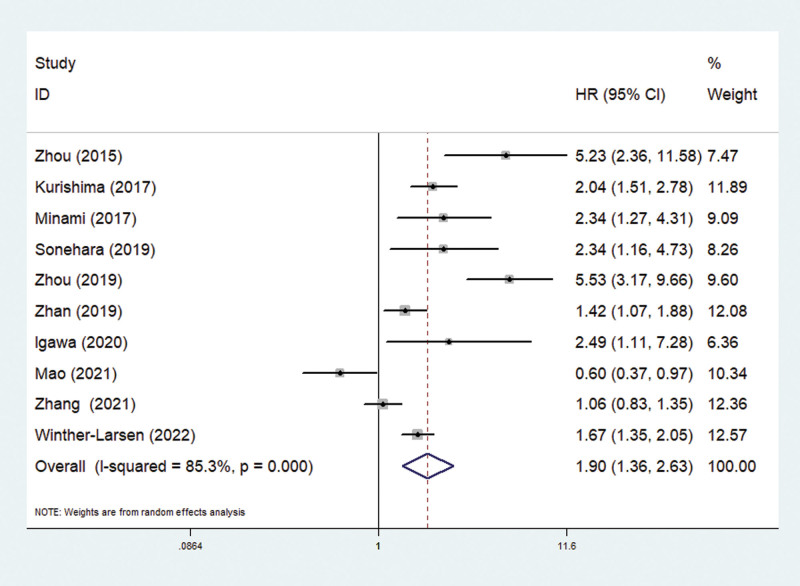
The association between pretreatment modified Glasgow Prognostic Score and overall survival in small cell lung cancer patients.

All included studies explored the predictive role of pretreatment mGPS for OS in SCLC.^[29–38]^ The pooled results manifested that an elevated pretreatment mGPS predicted poorer OS (HR = 1.90, 95% CI: 1.36–2.63, *P* < .001; *I*^2^ = 85.3%, *P* < .001) (Fig. 2).

Then subgroup analyses stratified by the country (China: HR = 1.81, 95% CI: 0.96–3.41, *P* = .065; Japan: HR = 2.15, 95% CI: 1.68–2.75, *P* < .001), tumor stage (mixed: HR = 1.77, 95% CI: 1.20–2.63, *P* = .004; extensive: HR = 2.37, 95% CI: 1.57–3.58, *P* < .001), treatment (mixed: HR = 2.66, 95% CI: 1.59–4.46, *P* < .001; chemotherapy: HR = 1.68, 95% CI: 1.00–2.82, *P* = .052) and comparison of mGPS (0 vs 1: HR = 1.31, 95% CI: 1.16–1.49, *P* < .001; 0 vs 2: HR = 2.75, 95% CI: 1.77–4.28, *P* < .001) manifested similar results, although the association between pretreatment mGPS and OS in Chinese patients and patients receiving the chemotherapy did not reach statistical difference (Table 2).

**Table 2:** Results of meta-analysis.

	No. of studies	HR	95% CI	*P* value	*I* ^2^	*P* value
Overall survival	10^[29–38]^	1.90	1.36–2.63	<.001	85.3	<.001
*Country*						
China	5^[29,33,34,36,37]^	1.81	0.96–3.41	.065	92.1	<.001
Japan	4^[30–32,35]^	2.15	1.68–2.75	<.001	0.0	.953
*Tumor stage*						
Mixed	7^[29,30,33,34,36–38]^	1.77	1.20–2.63	.004	89.5	<.001
Extensive	3^[31,32,35]^	2.37	1.57–3.58	<.001	0.0	.993
*Treatment*						
Mixed	3^[29,30,32]^	2.66	1.59–4.46	<.001	57.4	.096
Chemotherapy	6^[31,33–37]^	1.68	1.00–2.82	.052	88.7	<.001
*Comparison of mGPS*						
mGPS 0 vs 1	5^[29,30,32,33,38]^	1.31	1.16–1.49	<.001	0.0	.902
mGPS 0 vs 2	5^[29,30,32,33,38]^	2.75	1.77–4.28	<.001	81.3	<.001
Progression-free survival	3^[31,34,35]^	1.40	1.13–1.74	.002	0.0	.405

CI = confidence interval, HR = hazard ratio, mGPS = modified Glasgow Prognostic Score.

### 3.4. The association between pretreatment mGPS and PFS in SCLC patients

**Figure 3.**
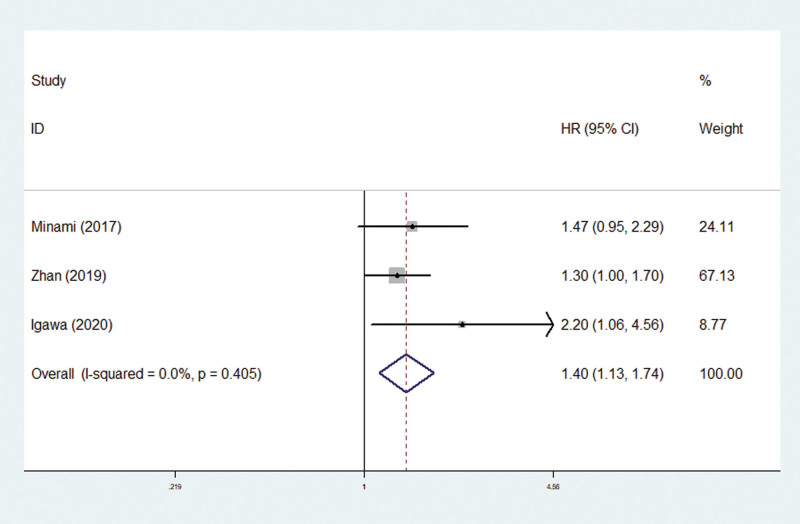
The association between pretreatment modified Glasgow Prognostic Score and progression-free survival in small cell lung cancer patients.

Only 3 studies explored the predictive role of pretreatment mGPS for PFS in SCLC patients.^[31,34,35]^ The pooled results demonstrated that pretreatment mGPS was significantly associated with PFS (HR = 1.40, 95% CI: 1.13–1.74, *P* = .002; *I*^2^ = 0.0%, *P* = .405) (Fig. 3) and patients with elevated mGPS experienced worse PFS.

### 3.5. Sensitivity analysis and publication bias

**Figure 4.**
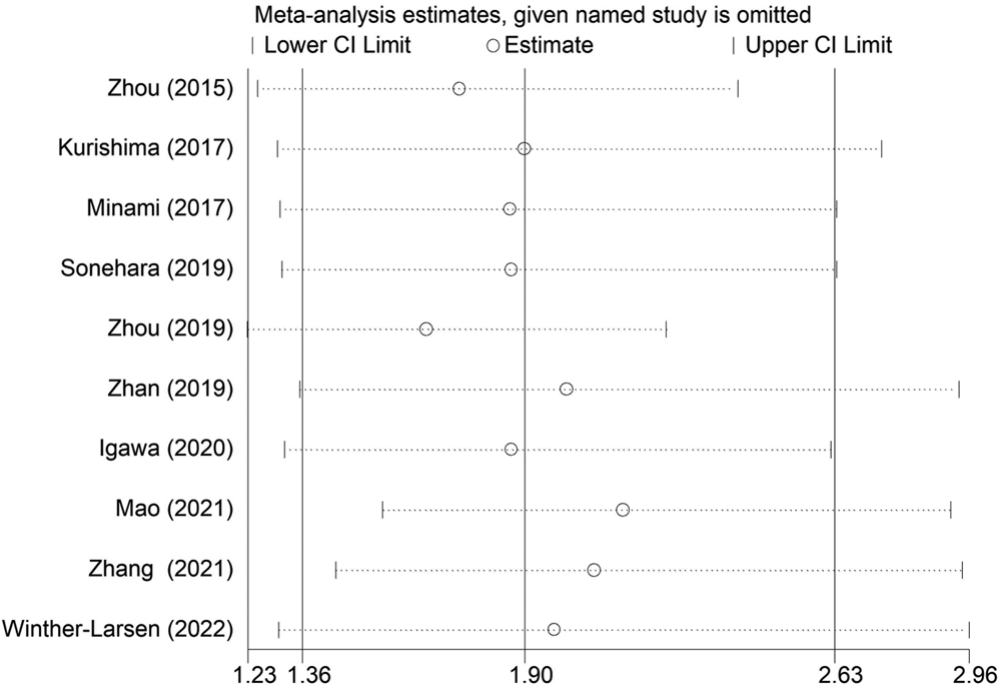
Sensitivity analysis about the association between pretreatment modified Glasgow Prognostic Score and overall survival in small cell lung cancer patients.

**Figure 5.**
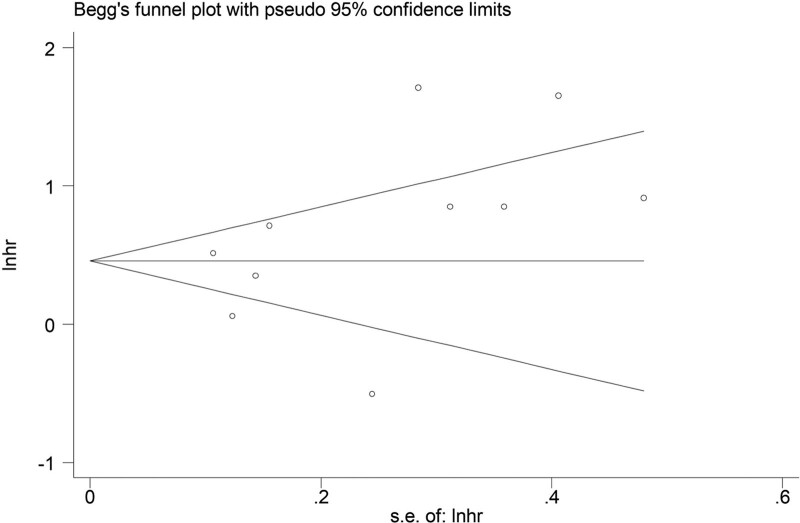
Begg funnel plot.

The sensitivity analysis was conducted by excluding each included studies one by one, which indicated that the results of this meta-analysis were stable and reliable (Fig. 4). Besides, according to the symmetrical Begg funnel plot (Fig. 5) and *P* = .198 in Egger test, no obvious publication bias was observed in our meta-analysis.

## 4. Discussion

The current meta-analysis demonstrated that pretreatment mGPS was significantly associated with survival in SCLC and patients with elevated mGPS had obviously poorer prognosis than patients with mGPS 0 did. Pretreatment mGPS could serve as a novel and reliable prognostic indicator and might contribute to the formulation of treatment strategy for SCLC patients. However, due to the limitations existed in included studies, more prospective randomized controlled trials (RCTs) are still needed to verify our results.

In the past decades, lots of researches have strongly indicated the significant relationship between inflammation and occurrence, development, and metastasis of tumors.^[39–41]^ In the early stages of the tumor, inflammatory mediators produced by inflammatory cells, such as cytokines, free radicals, prostaglandins, and growth factors, could induce genetic and epigenetic changes, including point mutations in tumor suppressor genes, DNA methylation, and posttranslational modifications, which leads to tumor development.^[42]^ Chronic inflammatory cells and inflammatory mediators contribute to the tumor microenvironment. Tumor microenvironment enables tumor cells not only to escape the immune surveillance of host, but also to become invasive due to the stimulation of some inflammatory medicators, and then these invasive tumor cells would invade surrounding tissues and neighboring organs, enter blood vessels and lymphatic vessels and spread to distant organs.^[43,44]^ Besides, inflammatory factors could also weaken the body’s immune function and further inhibit the body’s response to antitumor drugs.^[45]^ Therefore, the expression of inflammatory factors is important for the diagnosis and prognosis of cancer patients.

C-reactive protein is widely used as a marker of systematic inflammation and the elevation of C-reactive protein level is closely related to many pulmonary diseases including the pneumonia, malignancy, and pulmonary thromboembolism.^[46,47]^ Albumin is usually applied as a biomarker reflecting the nutritional status and decreased albumin concentration is closely associated with poor prognosis in cancer patients.^[48]^ Besides, it has been reported that albumin also plays a role in the systematic inflammation.^[49]^ In 2003, Forrest described the prognostic role of GPS in non-small cell lung cancer (NSCLC) for the first time.^[50]^ Then McMillan introduced the mGPS which showed higher value in predicting prognosis of cancer patients.^[51]^

Predictive role of mGPS for survival has been reported in several types of cancers. Tan et al included 13 studies involving 12,524 urothelial carcinoma patients and demonstrated that a high mGPS was obviously associated with poor OS (mGPS 0/1: HR = 1.33, *P* = .001; mGPS 0/2: HR = 2.02, *P* < .001) and PFS (mGPS 0/1: HR = 1.26, *P* = .021; mGPS 0/2: HR = 1.76, *P* = .013), recurrence-free survival (RFS) (mGPS 0/1: HR = 1.36, *P* < .001; mGPS 0/2: HR = 1.70, *P* < .001) and cancer-specific survival (mGPS 0/2: HR = 1.81, *P* < .001).^[21]^ Besides, Wu et al also manifested that an elevated mGPS predicted poor OS in patients with pancreatic cancer (HR = 1.92, *P* = .002).^[20]^ Tong et al enrolled 2691 patients from 15 cohort studies and indicated that mGPS was significantly associated with OS of renal cell carcinoma (mGPS 0/1: HR = 2.64, *P* = .002; mGPS 0/2: HR = 3.75, *P* < .001).^[19]^

As for lung cancer, only 2 meta-analyses identified the predictive role of mGPS for survival.^[23]^ Eleven studies were included in the meta-analysis by Jin et al and their results showed that elevated mGPS was significantly related to poorer OS (mGPS 0/1: HR = 1.74, *P* = .001; mGPS 0/2: HR = 5.82, *P* = .003; mGPS 0/1-2: HR = 1.42, *P* = .002). However, in this meta-analysis only 3 studies focusing on SCLC patients were included and only the predictive role of mGPS for OS was investigated.^[23]^ Meanwhile, the other meta-analysis by Yang et al just focused on NSCLC patients.^[52]^ Due to the substantial differences in disease biological characteristics and malignant risk between SCLC and NSCLC, we believe that it is necessary to further identify prognostic value of pretreatment mGPS in SCLC, which contributes to the risk evaluation for SCLC patients. Notably, the study by Mao et al reported an opposite association between elevated mGPS and poor OS in the multivariate analysis combining the distance metastasis and mGPS.^[36]^ However, their results of univariate analysis were consistent with our findings, which indicated that the prognostic role of mGPS might be affected by other parameters.

In clinics, it is still too difficult to accurately predict the survival and therapeutic effects for SCLC due to its high malignancy and recurrence rate. Besides, the prognostic value of tumor stage (limited or extensive stage) is very limited in SCLC patients. According to our results, mGPS is verified to show high prognostic value in SCLC patients, predicting the overall prognosis and disease progression despite of the tumor stage and treatment. Therefore, mGPS might play a role in contributing to the formulation of treatment strategy and assessment of the disease progression.

Actually, there are still some field worthy of further investigations about the mGPS in SCLC. For example, it is unclear whether the change of mGPS during antitumor therapy contributes to the prediction of survival of SCLC patients. Besides, it is necessary to explore whether lowering the mGPS could improve prognosis of SCLC patients. Furthermore, a combination of mGPS and other prognostic factors might be more valuable and reliable in accurately predicting survival of SCLC patients.

There are several limitations in this meta-analysis. First, all included studies are retrospective with relatively small sample sizes, which may cause some bias. Second, most included studies are from China and Japan, which affects the generalizability of the conclusion. Third, obvious heterogeneity existed among included studies. However, we could not well identify main sources of heterogeneity based on the results of subgroup analysis. Four, due to the lack of original data, we were unable to conducted more detailed subgroup analysis based on other important parameters such as the age and comorbidity.

## 5. Conclusion

Pretreatment mGPS was significantly associated with prognosis in SCLC and patients with elevated mGPS experienced obviously worse survival. Thus, pretreatment mGPS could serve as a novel and reliable prognostic indicator in SCLC patients. However, more prospective high-quality studies are still needed to verify above findings.

## Author contributions

**Conceptualization:** Yulian Xie, Yang Hu.

**Data curation:** Hongjun Li.

**Formal analysis:** Yulian Xie, Hongjun Li.

**Investigation:** Hongjun Li.

**Methodology:** Yulian Xie.

**Resources:** Hongjun Li.

**Software:** Yulian Xie, Hongjun Li.

**Supervision:** Yang Hu.

**Writing – original draft:** Yulian Xie.

**Writing – review & editing:** Yang Hu.
